# Effect of high-intensity training on improving knee flexion strength and quality of life in patients with knee osteoarthritis: a systematic review and meta-analysis of randomized controlled trials

**DOI:** 10.3389/fphys.2025.1561697

**Published:** 2025-06-27

**Authors:** Bo Zhai, Yu Wang, Zhongliang Zhang, Qiang Wang

**Affiliations:** ^1^ Department of Joint Surgery, JINHUA Hospital of Traditional Chinese Medicine, Jinhua, Zhejiang, China; ^2^ Department of Trauma One, JINHUA Hospital of Traditional Chinese Medicine, Jinhua, Zhejiang, China

**Keywords:** high-intensity, exercise, knee osteoarthritis, RCTs, meta-analysis

## Abstract

**Objectives:**

Whether high-intensity exercise can effectively improve the symptoms and quality of life of patients with knee osteoarthritis has not been determined. This study updates the evidence on the efficacy of high-intensity training for patients with knee osteoarthritis by integrating all large-scale randomized controlled trials (RCTs).

**Methods:**

A systematic literature search was conducted in PubMed, Embase, Web of Science, and Cochrane through November 2024. Outcomes assessed included Western Ontario and McMaster Universities Osteoarthritis Index (WOMAC), 6-min walk test (6-MWT), knee flexion strength, knee extension strength, leg press strength, Knee Injury and Osteoarthritis Outcome Score (KOOS) (pain, symptoms, and QoL), stair climbing test, and timed-up-and-go (TUG). Standardized mean differences (SMD) with 95% confidence intervals (CI) were used to pool continuous variables. Sensitivity analysis was performed to assess result stability. Analyses were conducted using Review Manager 5.4 and STATA 15.1.

**Results:**

Nine RCTs with 1,540 patients were included. The average age of the patients ranged from 59.1 to 69 years. Meta-analysis showed significant improvements in knee flexion strength (SMD: 0.39; 95% CI: 0.08, 0.70), leg press strength (SMD: 0.47; 95% CI: 0.23, 0.70), KOOS symptoms (SMD: 0.18; 95% CI: 0.02, 0.35), and KOOS QoL (SMD: 0.29; 95% CI: 0.13, 0.45) in the high-intensity exercise group compared to the control. However, high-intensity exercise had no significant effect on WOMAC, 6-MWT, knee extension strength, KOOS pain, stair climbing, or TUG.

**Conclusion:**

High-intensity exercise significantly improves knee flexion strength, leg press strength, and KOOS symptoms and QoL in knee osteoarthritis patients. Given the study’s limitations, further large-scale, multicenter RCTs are needed to confirm the rehabilitation effects and potential influencing factors of high-intensity exercise.

**Systematic Review Registration:**

https://www.crd.york.ac.uk/PROSPERO/

## 1 Introduction

Chronic knee osteoarthritis is prevalent in middle-aged and elderly individuals. With an aging population, its prevalence and incidence are increasing, and it carries a 53% disability rate, contributing significantly to the economic burden (Cross et al., 2014; O’Sullivan, 2024). The main symptoms include knee pain, stiffness, functional impairment, and limited mobility (Lo et al., 2018). Reduced physical activity accelerates age-related muscle loss, particularly in the knee area, leading to sarcopenia (Sayer et al., 2013). Weakened muscle strength around the knee and increased cartilage wear can trigger symptoms such as inflammation and joint instability, raising the risk of obesity and metabolic syndrome. This creates a vicious cycle, exacerbating the patient’s condition and ultimately leading to immobility, disability, and health complications (Yoshimura et al., 2012). The American Academy of Orthopedic Surgeons (AAOS) recommends strength training, aerobic exercise, adherence to national physical activity guidelines, and weight loss to manage knee osteoarthritis (Jones et al., 2015). Systematic reviews and meta-analyses (Tanaka et al., 2015; Uthman et al., 2014) have shown that exercise improves body composition, physical function, metabolic health, and quality of life in middle-aged and elderly patients with knee osteoarthritis. Exercise regimens typically involve medium to low-intensity strength training, aerobic training, and sessions lasting 30–60 min. These routines can be monotonous, making adherence challenging for many patients. Recent studies suggest that high-intensity training may be more effective than moderate or low-intensity options (Casaña et al., 2019; Keogh et al., 2017).

Currently, two common exercise interventions for improving mobility and aerobic capacity are moderate-intensity training and high-intensity training. Moderate-intensity training primarily involves moderate-intensity continuous training (MICT), which consists of continuous exercise at 64%–75% of maximum heart rate (HRmax) or 40%–59% of reserve heart rate (HRR). The most common form of high-intensity training is high-intensity interval training (HIIT), which alternates between brief, intense bursts and longer recovery periods on a shorter interval basis (Boyne et al., 2016). Studies have shown that HIIT outperforms low- and medium-intensity exercise in improving aerobic capacity and other functions in healthy adults and patients with cardiovascular diseases (Chen et al., 2024; Costache et al., 2024; Oliveira et al., 2024; Wang and Wang, 2024). High-intensity exercise is now widely used in the prevention and treatment of chronic conditions such as overweight/obesity, cardiovascular disease, cancer, and type 2 diabetes. It enhances aerobic fitness, insulin sensitivity, and chronic disease management. Additionally, it significantly impacts body fat, blood biomarkers, body composition, and skeletal muscle activation. Its safety, effectiveness, and benefits have been well established (Buchheit and Laursen, 2013; Ross et al., 2016). Research on the use of high-intensity exercise for rehabilitation in patients with chronic knee joint diseases is growing, but the optimal exercise prescription, target groups, and effects remain unclear (Husby et al., 2018; Roxburgh et al., 2024; Thudium et al., 2023).

The meta-analysis by Hua et al. (2023) included randomized controlled trials (RCTs) published before March 2021. The results showed that high-intensity strength training had effects similar to low-intensity training and usual care in improving knee pain, knee function, and quality of life. However, limitations such as small sample sizes, unexplained heterogeneity, and flaws in study design reduced the quality of evidence in their analysis (Hua et al., 2023). More recently, several large, well-designed randomized controlled trials have reported on the rehabilitation effects of high-intensity exercise for knee osteoarthritis, potentially altering the current understanding of its benefits for this condition (Su et al., 2024; Torstensen et al., 2023; de Zwart et al., 2022; Messier et al., 2021). However, the four recently published large RCTs did not reach completely consistent conclusions. Three of the studies found that high-intensity exercise had no significant advantage in improving the symptoms of patients with knee osteoarthritis (Su et al., 2024; de Zwart et al., 2022; Messier et al., 2021). However, the other one study found that high-intensity exercise was significantly more effective for the improvement of quality of life than low-intensity exercise for patients with knee osteoarthritis (Torstensen et al., 2023). Therefore, this article aims to conduct an updated systematic review and meta-analysis of high-quality randomized controlled trials, clarify the clinical benefits and limitations of high-intensity training for patients with knee osteoarthritis by integrating new evidence, and evaluate the exact effect of high-intensity exercise on the rehabilitation of knee osteoarthritis. This study hypothesized that high-intensity training would be superior to conventional rehabilitation in terms of improving strength and quality of life.

## 2 Methods

### 2.1 Literature search

This meta-analysis was conducted according to the PRISMA 2020 guidelines (Page et al., 2021) and registered in PROSPERO (CRD42024633711). We performed a systematic literature search in PubMed, Embase, Web of Science, and Cochrane up to November 2024 for RCTs comparing high-intensity exercise with routine rehabilitation for knee osteoarthritis. The search terms included “exercise,” “knee osteoarthritis,” and “high-intensity.” The detailed PubMed search strategy was: ((“Exercise”[Mesh]) OR (Exercises OR Physical Exercise OR Physical Activity OR Aerobic Exercise OR Isometric Exercises OR Acute Exercise OR Exercise Training)) AND (high-intensity)) AND ((“Osteoarthritis, Knee”[Mesh]) OR (Knee Osteoarthritides OR Knee Osteoarthritis)) AND (Random*). We also manually screened the reference lists of included RCTs. Two authors independently retrieved and assessed eligible articles, resolving any discrepancies through discussion. The full search strategy is presented in Supplementary Table S1.

### 2.2 Inclusion and exclusion criteria

Articles were eligible if they met the following criteria:

P: Patients diagnosed with knee osteoarthritis.
I: High-intensity exercise.
C: Routine rehabilitation, including moderate or low-intensity exercise, or no exercise intervention.
O: Outcomes such as the Western Ontario and McMaster Universities Osteoarthritis Index (WOMAC), 6-min walk test (6-MWT), knee flexion strength, knee extension strength, leg press strength, Knee Injury and Osteoarthritis Outcome Score (KOOS) (pain, symptoms, and quality of life (QoL)), stair climbing test, timed-up-and-go (TUG), etc.
S: RCTs.

We excluded study protocols, unpublished studies, non-original studies (including meeting abstracts, corrections, and replies), non-RCT studies, studies without sufficient data, and reviews.

### 2.3 Data abstraction

Two authors independently performed data abstraction, resolving any discrepancies with a third author. The following data were extracted from eligible RCTs: first author name, publication year, study region, study design, registration number, intervention, control, sample size, age, gender, intervention duration, WOMAC, 6-MWT, knee flexion strength, knee extension strength, leg press strength, KOOS (pain, symptoms, and QoL), stair climbing test, and TUG. If data were insufficient, the corresponding authors were contacted to obtain complete data.

### 2.4 Quality evaluation

The quality of eligible RCTs was assessed according to the Cochrane Handbook for Systematic Reviews of Interventions 5.1.0, evaluating seven domains: sequence generation randomization, allocation concealment, blinding of participants and personnel, outcome assessment blinding, incomplete outcome data, selective outcome reporting, and other potential sources of bias (Cumpston et al., 2019). Each study was assigned one of three risk ratings: low, high, or unclear. Studies with more “low-risk” evaluations were considered of higher quality. Two authors independently assessed the quality of all included studies and resolved any disagreements through discussion.

### 2.5 Statistical analysis

Data synthesis was conducted using Review Manager 5.4.1. For continuous outcomes, standardized mean differences (SMD) with 95% confidence intervals (CI) were used. Heterogeneity across outcomes was assessed using the chi-squared (χ^2^) test (Cochran’s Q) and the inconsistency index (Higgins and Thompson, 2002). Substantial heterogeneity was defined by a χ^2^ P value < 0.1 or an I^2^ > 50%. The overall SMD was calculated using the random-effects model. For outcomes with more than two studies and significant heterogeneity, a sensitivity analysis was performed to assess the impact of each individual RCT on the overall SMD. Publication bias was evaluated using funnel plots and Egger’s regression tests (Egger et al., 1997) in Stata 15.1 (Stata Corp, College Station, Texas, United States) for outcomes with more than ten studies. A P value < 0.05 was considered indicative of statistically significant publication bias.

## 3 Results

### 3.1 Literature retrieval, study characteristics, and baseline

**FIGURE 1:**
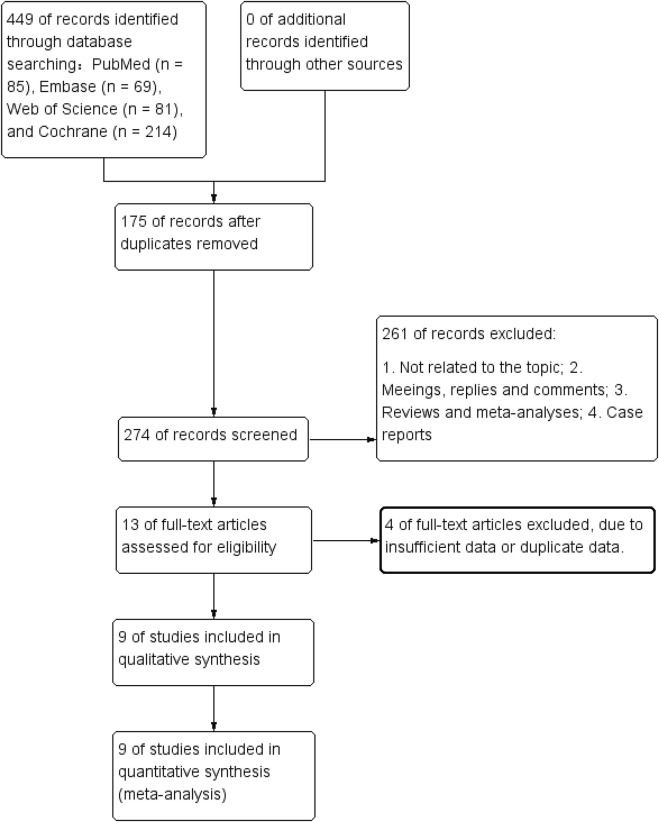
Flowchart of the systematic search and selection process.

**FIGURE 2:**
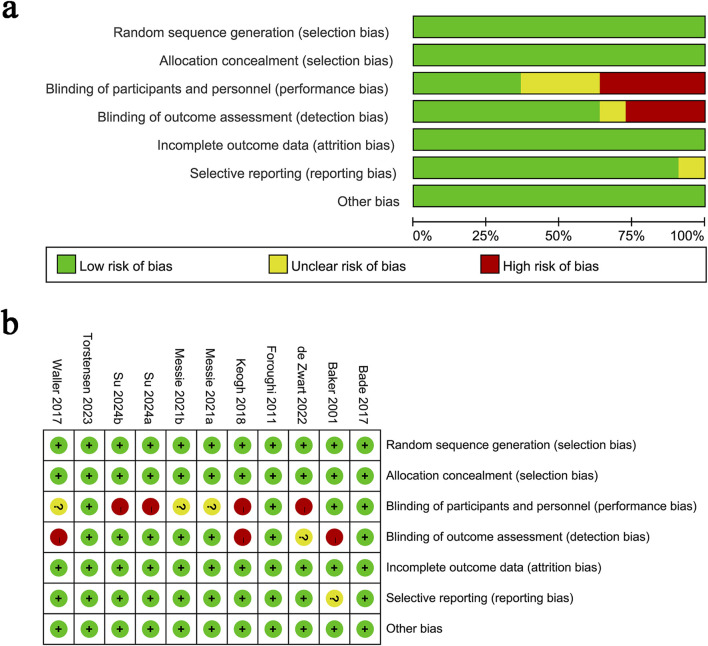
Details of the quality evaluation for included RCTs. **(a)** Risk bias of summary; **(b)** Risk bias of graph.

Figure 1 presents the flowchart of the literature retrieval and selection process. A total of 449 studies were identified from PubMed (n = 85), Embase (n = 69), Web of Science (n = 81), and Cochrane (n = 214) through systematic literature search. After removing duplicates, 274 titles and abstracts were screened. Ultimately, 9 RCTs (Su et al., 2024; Torstensen et al., 2023; de Zwart et al., 2022; Messier et al., 2021; Bade et al., 2017; Waller et al., 2017; Baker et al., 2001; Keogh et al., 2018; Foroughi et al., 2011) with 1,540 patients were included in the meta-analysis. Table 1 lists the characteristics of the included RCTs. The quality evaluation of all included RCTs is shown in Figure 2. The definition of “high intensity” in various studies is mainly based on one or more of the following methods (Cross et al., 2014): subjective rating of exertion (RPE, ≥7/10 or ≥15/20) (O’Sullivan, 2024); percentage of maximum repetitions (%1RM, ≥70–80%); (Lo et al., 2018); percentage of maximum heart rate (%HRmax, ≥80%) (Sayer et al., 2013); percentage of heart rate reserve (%HRR, ≥75%) (Yoshimura et al., 2012); reaching or approaching exhaustion (Jones et al., 2015); based on the maximum intensity tolerated by the individual. Training forms vary, including machine resistance training (such as leg press, knee flexion and extension machine), circuit training, bicycle interval training, water resistance training, and progressive training at home. Training frequency is mostly 2–3 times a week, and the duration of a single training session (excluding warm-up and cool-down) is usually between 20–60 min. The duration of the “working” period of high-intensity interval training varies from 30 s to 4 min, and the rest interval is mostly 45–90 s or active recovery (low intensity) is used.

**TABLE 1:** Characteristics of included studies.

Study	Region	Study design	Intervention	Intervention time	Control	Sample size		Mean/median age		No. of male		Mean/median BMI	
						High-intensity	Control	High-intensity	Control	High-intensity	Control	High-intensity	Control
Bade 2017	United States	RCT	High-intensity progressive rehabilitation	11 weeks	Low-intensity rehabilitation	84	78	63	64	39 (46.4%)	34 (43.6%)	31	30
Baker 2001	United States	RCT	Home based progressive strength training program	4 months	Nutrition education program	23	23	69	68	6 (26.1%)	4 (17.4%)	31	32
de Zwart 2022	Netherlands	RCT	High-intensity resistance training	12 weeks	Low-intensity resistance training	89	88	67.3	67.9	32 (36.0%)	38 (43.2%)	28.6	27.7
Foroughi 2011	Australia	RCT	High intensity progressive resistance training	6 months	Sham-exercise program	26	28	66	65	0	0	31.4	32.7
Keogh 2018	Australia	RCT	High-intensity interval training	8 weeks	Moderate-intensity continuous training	9	8	59.1	66.1	3 (33.3%)	1 (12.5%)	27	28.2
Messie 2021a	United States	RCT	High-intensity strength training	18 months	Low-intensity strength training	127	126	67	64	75 (59.1%)	75 (59.5%)	31	31
Messie 2021b	United States	RCT	High-intensity strength training	18 months	Attention control	127	124	67	64	75 (59.1%)	76 (61.3%)	31	32
Su 2024a	China	RCT	Threshold training/high-intensive stationary cycling training	6 months	Intensive endurance/moderate-intensive stationary cycling training	76	77	61.4	61.7	36 (47.4%)	37 (48.1%)	30.8	30.3
Su 2024b	China	RCT	Threshold training/high-intensive stationary cycling training	6 months	Regular rehabilitation programs	76	75	61.4	61.2	36 (47.4%)	39 (52%)	30.8	30.1
Torstensen 2023	Sweden	RCT	High-dose exercise therapy	12 weeks	Low-dose exercise therapy	98	91	63.1	62	41 (41.8%)	42 (46.2%)	27.8	28.1
Waller 2017	Finland	RCT	Supervised intensive aquatic resistance training sessions	4 months	Normal physical activity	43	44	63.8	63.9	0	0	26.6	27.1

### 3.2 Effects of high-intensity exercise on improving muscle strength

#### 3.2.1 WOMAC

**FIGURE 3:**
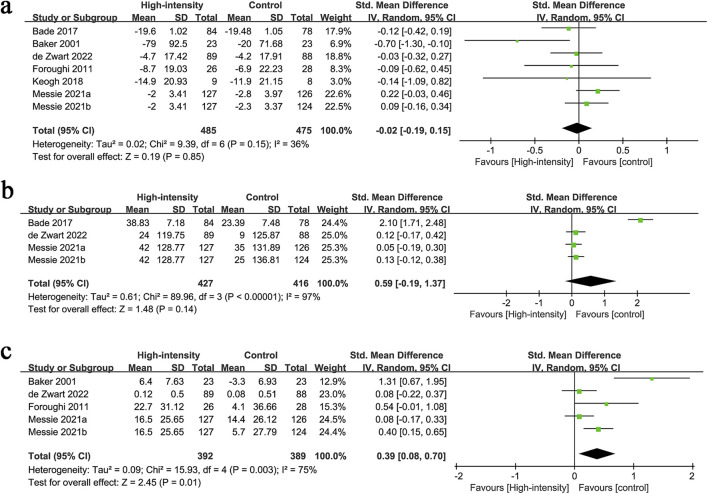
Forest plots of **(a)** WOMAC, **(b)** 6-MWT, **(c)** knee flexion strength.

WOMAC results were synthesized from 7 RCTs with 960 patients. The meta-analysis revealed no significant difference in WOMAC changes between the two groups (SMD: −0.02; 95% CI: −0.19, 0.15; P = 0.85), with no significant heterogeneity (I^2^ = 36%, P = 0.15) (Figure 3a).

#### 3.2.2 6-MWT

6-MWT results were synthesized from 4 RCTs with 843 patients. The meta-analysis revealed no significant difference in 6-MWT changes between the two groups (SMD: 0.59; 95% CI: −0.19, 1.37; P = 0.14), with significant heterogeneity (I^2^ = 97%, P < 0.00001) (Figure 3b).

#### 3.2.3 Knee flexion strength

Knee flexion strength results were synthesized from 5 RCTs with 781 patients. The meta-analysis revealed a significantly higher increment in knee flexion strength in the high-intensity exercise group (SMD: 0.39; 95% CI: 0.08, 0.70; P = 0.01), with significant heterogeneity (I^2^ = 75%, P = 0.003) (Figure 3c).

#### 3.2.4 Knee extension strength

**FIGURE 4:**
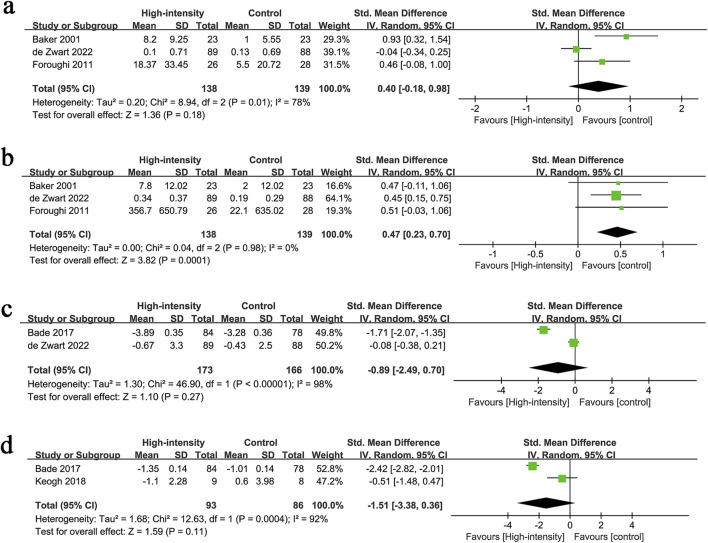
Forest plots of **(a)** knee extension strength, **(b)** leg press strength, **(c)** stair climbing test, **(d)** TUG.

Knee extension strength results were synthesized from 3 RCTs with 277 patients. The meta-analysis revealed no significant difference in knee extension strength changes between the two groups (SMD: 0.40; 95% CI: −0.18, 0.98; P = 0.18), with significant heterogeneity (I^2^ = 78%, P = 0.01) (Figure 4a).

#### 3.2.5 Leg press strength

Leg press strength results were synthesized from 3 RCTs with 277 patients. The meta-analysis revealed a significantly higher increment in leg press strength in the high-intensity exercise group (SMD: 0.47; 95% CI: 0.23, 0.70; P = 0.0001), with no significant heterogeneity (I^2^ = 0%, P = 0.98) (Figure 4b).

#### 3.2.6 Stair climbing test

Stair climbing test results were synthesized from 2 RCTs with 339 patients. The meta-analysis revealed no significant difference in stair climbing performance between the two groups (SMD: −0.89; 95% CI: −2.49, 0.70; P = 0.27), with significant heterogeneity (I^2^ = 98%, P < 0.00001) (Figure 4c).

#### 3.2.7 TUG

TUG results were synthesized from 2 RCTs with 179 patients. The meta-analysis revealed no significant difference in TUG performance between the two groups (SMD: −1.51; 95% CI: −3.38, 0.36; P = 0.11), with significant heterogeneity (I^2^ = 92%, P = 0.0004) (Figure 4d).

### 3.3 Effect of high-intensity exercise on improving patients’ quality of life

#### 3.3.1 KOOS pain

**FIGURE 5:**
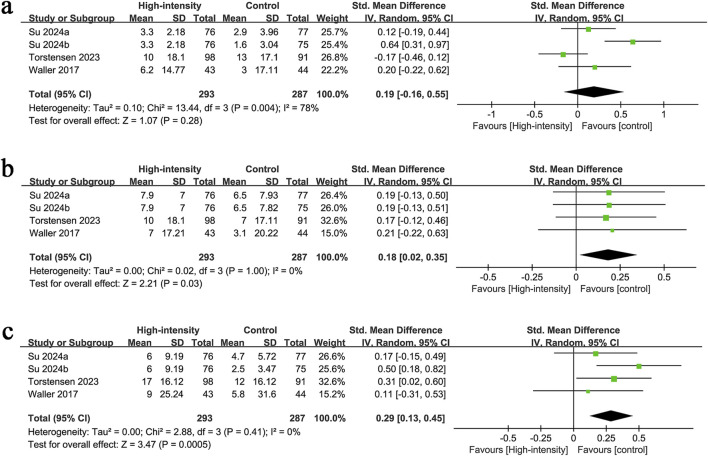
Forest plots of **(a)** KOOS pain, **(b)** KOOS symptoms, **(c)** KOOS QoL.

KOOS pain results were synthesized from 4 RCTs with 580 patients. The meta-analysis revealed no significant difference in KOOS pain changes between the two groups (SMD: 0.19; 95% CI: −0.16, 0.55; P = 0.28), with significant heterogeneity (I^2^ = 78%, P = 0.004) (Figure 5a).

#### 3.3.2 KOOS symptoms

KOOS symptoms results were synthesized from 4 RCTs with 580 patients. The meta-analysis revealed a significantly higher increment in KOOS symptoms in the high-intensity exercise group (SMD: 0.18; 95% CI: 0.02, 0.35; P = 0.03), with no significant heterogeneity (I^2^ = 0%, P = 1.00) (Figure 5b).

#### 3.3.3 KOOS QoL

KOOS QoL results were synthesized from 4 RCTs with 580 patients. The meta-analysis revealed a significantly higher increment in KOOS QoL in the high-intensity exercise group (SMD: 0.29; 95% CI: 0.13, 0.45; P = 0.0005), with no significant heterogeneity (I^2^ = 0%, P = 0.41) (Figure 5c).

### 3.4 Sensitivity analysis

Given the significant heterogeneity, we conducted a sensitivity analysis for the results of 6-MWT, knee flexion strength, knee extension strength, and KOOS pain to assess the impact of each RCT on the overall SMD by sequentially excluding eligible RCTs. The sensitivity analysis showed that the total SMD remained stable after excluding each RCT for 6-MWT (Supplementary Figure S1A) and KOOS pain (Supplementary Figure S1B). However, excluding Messier et al. (2021) caused the significant difference in knee flexion strength to become nonsignificant (SMD: 0.41; 95% CI: −0.01, 0.83), indicating substantial instability in this outcome (Supplementary Figure S1C). Additionally, excluding de Zwart et al. (2022) caused the nonsignificant difference in knee extension strength to become significant (SMD: 0.67; 95% CI: 0.21, 1.13), suggesting considerable instability in this outcome (Supplementary Figure S1D). Sensitivity analysis also identified the main sources of heterogeneity: Bade et al. (2017) for 6-MWT, Baker et al. (2001) for knee flexion strength, de Zwart et al. (2022) for knee extension strength, and Su et al. (2024) for KOOS pain.

## 4 Discussion

International guidelines recommend managing knee osteoarthritis through the promotion of physical activity and regular exercise participation (Lane et al., 2015). Dunlop et al. (2019) and Dunlop et al. (2014) demonstrated that 1 h of moderate-intensity vigorous activity per week reduces the disability rate in knee osteoarthritis, with risk decreasing as activity levels increase. Although MICT rehabilitation has been traditionally recommended for knee osteoarthritis patients, recent studies suggest that high-intensity exercise may offer greater benefits (Keogh et al., 2018). Previous meta-analyses did not show a significant advantage of high-intensity exercise, which contrasts with recent findings from high-quality RCTs. However, the latest RCT results of Torstensen et al. suggest that high-intensity exercise is significantly superior to low-intensity exercise in improving the quality of life of patients with knee osteoarthritis (Torstensen et al., 2023). This study aims to conduct an updated systematic review and meta-analysis of all published high-quality randomized controlled trials to evaluate the precise effect of high-intensity exercise on the rehabilitation of knee osteoarthritis patients.

This study found that high-intensity exercise significantly improved knee flexion strength, leg press strength, KOOS symptoms, and KOOS QoL compared to conventional rehabilitation. These results suggest that incorporating high-intensity exercise into conventional rehabilitation enhances lower limb muscle and joint strength, while improving clinical symptoms and overall quality of life, aligning with the conclusions of recent RCTs (Su et al., 2024; Torstensen et al., 2023; de Zwart et al., 2022; Messier et al., 2021). However, the conclusions of this study differ from those of the meta-analysis by Hua et al. (2023), which found that high-intensity strength training did not improve muscle strength or symptoms compared to low-intensity training and conventional rehabilitation. This discrepancy is primarily due to differences in sample size and study quality. This study included a larger sample and higher-quality RCTs with strong design and complete follow-up, resulting in superior evidence quality.

This meta-analysis found no significant advantage of high-intensity exercise in improving WOMAC, 6-MWT, knee extension strength, KOOS pain, stair climbing test, and TUG in patients with knee osteoarthritis, consistent with the findings of Hua et al. (2023). However, sensitivity analysis revealed significant instability in knee extension strength. Excluding the data of de Zwart et al. (2022) changed the result from nonsignificant to significant and reduced the heterogeneity from 78% to 21%. This suggests that high-intensity exercise may have some effect on knee extension strength, but further large-scale RCTs are needed for confirmation. In conclusion, although high-intensity exercise has limited efficacy in improving disease-specific symptoms and cardiopulmonary function in knee osteoarthritis patients, the small number of studies and limited evidence quality highlight the need for further research to validate these findings. These analyses show that the effects of high-intensity training on different outcomes in patients with knee osteoarthritis are heterogeneous and are significantly affected by the characteristics of the study population (including whether they are postoperative and whether they have comorbidities), the details of the specific intervention program (including the intensity definition threshold, training mode, degree of supervision, and period length), and the type of control group. These factors must be considered when interpreting the results of this meta-analysis and designing future research or clinical programs. Future studies need to report and standardize the definition of “high intensity” more accurately and conduct in-depth exploration of specific subpopulations and specific intervention models.

Historically, high-intensity training prescriptions included warm-up exercises, strength training, interval recovery, and relaxation (Gil et al., 2014). Patients with knee osteoarthritis can engage in various forms of exercise, such as running (Bartlett et al., 2018), walking (Fenton et al., 2018), machine exercise (Casaña et al., 2019; Bade and Stevens-Lapsley, 2011), freehand exercise (Casaña et al., 2019), cycling (Thomsen et al., 2018), and aquatic training (de Ruiter et al., 2017). These exercises typically last 6–12 weeks (Keogh et al., 2018; Thomsen et al., 2018), with 2–4 sessions per week, and consist of multiple short bursts of high-intensity training (30 s–4 min) in each session. An appropriate interval of 45–90 s is recommended between training sessions, which can be adjusted based on the patient’s condition. During this interval, low- to medium-intensity exercises or complete rest are performed. The total exercise time is typically short, not exceeding 30 min. Exercise intensity is primarily based on the patient’s subjective tolerance of maximum intensity (Bade and Stevens-Lapsley, 2011). Aquatic training programs effectively reduce the load on the knee joint but are challenging to implement in clinical settings and are costly. Machine exercises require specialized equipment and professional guidance. In contrast, running, walking, and freehand exercises are easy to perform and are not limited by environmental or time constraints. However, home-based exercise programs often lack objective data on the frequency, duration, and intensity of exercise performed by participants, which hinders the quantification of the dose-response relationship and the evaluation of feasibility and efficacy. Future studies could incorporate tools such as heart rate monitors and cycle ergometers to objectively record exercise intensity.

The safety of high-intensity training has been demonstrated in other chronic diseases (Hussain et al., 2016), but there is limited research on its effects on musculoskeletal health, muscle mass, strength, or function in patients with chronic arthritis (Sandstad et al., 2015). One factor contributing to this research gap is the concern about the safety of high-intensity exercise for patients with knee disease, particularly when performed unsupervised at home or in a fitness center. High-intensity exercise may result in musculoskeletal issues such as muscle strain, joint pain, and swelling (Liu and Latham, 2010). However, Sandstad et al. (2015) found no exercise-related adverse effects in a 10-week high-intensity interval training (HIIT) program. Bade et al. (2017) reported that high-intensity training was safe and feasible for patients undergoing total knee replacement surgery, with no muscle damage observed. Conversely, Regnaux et al. (2015) noted that 17 out of 1,000 participants reported adverse effects associated with high-intensity exercise, with nearly 2% experiencing adverse reactions, although no serious events were recorded. Keogh et al. (2018) reported adverse events in three out of 27 patients (one in the MICT group and two in the HIIT group) during an 8-week program of four training sessions per week. A total of 28 adverse event reports were made, with 24 attributed to one HIIT participant. These findings suggest that, even with strict inclusion criteria, a small number of patients may still experience adverse effects, making the safety of HIIT in patients with knee osteoarthritis a subject of ongoing debate. Future research should focus on determining whether patients with knee osteoarthritis who retain ambulatory function can benefit significantly from high-intensity training.

## 5 Clinical implications

Based on the results of this study, clinicians should encourage patients with knee osteoarthritis who retain walking function to participate in structured high-intensity training to improve muscle strength and quality of life. This study found that high-intensity training can significantly improve knee flexion strength and leg press strength, and improve KOOS symptoms and quality of life. For patients without obvious joint deformity (Kellgren-Lawrence grade ≤ III), BMI < 30, and no severe cardiovascular disease, a progressive structured program is recommended: initially, equipment resistance training is performed under the supervision of a rehabilitation therapist, and gradually transitions to home training. Pain response should be monitored during training, and the load should be adjusted every 4 weeks to avoid a plateau effect. It is worth noting that although high-intensity training did not significantly improve pain scores and cardiopulmonary function, its advantages in delaying muscle attenuation and maintaining daily activity ability make it a supplementary program for traditional low- and medium-intensity rehabilitation. In clinical practice, high-intensity resistance training can be combined with neuromuscular control exercises to form a synergistic intervention model to optimize long-term prognosis. In the future, it is necessary to further develop stratified training guidelines to clarify the intensity thresholds for patients at different stages of the disease course.

## 6 Limitations and strength

We must acknowledge several limitations of this meta-analysis. First, due to the inherent limitations of exercise intervention studies, only 36% of the nine included RCTs had a low risk of blinding of participants and personnel (performance bias), which may somewhat affect the reliability of the evidence. Second, the included RCTs varied in intervention measures (e.g., different high-intensity exercise methods, durations, and frequencies), which could introduce potential heterogeneity. Third, due to the limited number of studies, this meta-analysis was unable to analyze the effects of high-intensity exercise on other factors such as psychological state and motor function in patients with knee osteoarthritis, which warrants further research. Additionally, due to data limitations, subgroup analyses based on intervention methods, intensity, duration, frequency, age, race, and other factors were not feasible, preventing us from determining how these variables might influence the results. Fourth, most of the included studies were from European and American countries, with limited data from regions such as Asia and Africa. Therefore, the generalizability of these findings to global populations remains uncertain. Besides, the control groups included in the studies included low-intensity training, routine care, and no intervention, which may affect the extrapolation of the results. It is recommended that future studies adopt a standardized control design. At the same time, considering that most of the original studies included in this meta-analysis are short-term intervention experiments, more RCTs with longer intervention periods (such as more than 12 weeks or half a year) are needed in the future to observe the long-term effects of continuous training. Furthermore, future studies should collect or include more objective indicators (such as imaging, gait analysis, inflammatory biomarkers, etc.) to evaluate the rehabilitation effects of high-intensity training from multiple perspectives. Despite these limitations, this updated meta-analysis of high-quality RCTs overcomes previous challenges, such as small sample sizes and study design flaws. It confirms the effectiveness of high-intensity exercise for the rehabilitation of knee osteoarthritis patients and provides valuable, evidence-based insights for clinical exercise rehabilitation.

## 7 Conclusion

This study found that high-intensity exercise significantly improves knee flexion strength, leg press strength, KOOS symptoms, and KOOS QoL in patients with knee osteoarthritis. However, it had no significant effect on WOMAC, 6-MWT, knee extension strength, KOOS pain, stair climbing test, or TUG. Clinicians should encourage knee osteoarthritis patients with preserved walking function to engage in structured high-intensity training to enhance muscle strength and quality of life. Given the limitations of this study, including a small number of included studies, regional selection bias, and potential outcome instability, further large-sample, multicenter RCTs are needed to confirm the rehabilitation effects of high-intensity exercise and identify influencing factors in this patient population.

## Data Availability

The original contributions presented in the study are included in the article/Supplementary Material, further inquiries can be directed to the corresponding author.

## References

[B1] Bade M. J. Stevens-Lapsley J. E. (2011). Early high-intensity rehabilitation following total knee arthroplasty improves outcomes. J. Orthop. and Sports Phys. Ther. 41 (12), 932–941. 10.2519/jospt.2011.3734 21979411 PMC13040458

[B2] Bade M. J. Struessel T. Dayton M. Foran J. Kim R. H. Miner T. (2017). Early high-intensity *versus* low-intensity rehabilitation after total knee arthroplasty: a randomized controlled trial. Arthritis Care and Res. 69 (9), 1360–1368. 10.1002/acr.23139 PMC5415445 27813347

[B3] Baker K. R. Nelson M. E. Felson D. T. Layne J. E. Sarno R. Roubenoff R. (2001). The efficacy of home based progressive strength training in older adults with knee osteoarthritis: a randomized controlled trial. J. Rheumatology 28 (7), 1655–1665. 11469475

[B4] Bartlett D. B. Willis L. H. Slentz C. A. Hoselton A. Kelly L. Huebner J. L. (2018). Ten weeks of high-intensity interval walk training is associated with reduced disease activity and improved innate immune function in older adults with rheumatoid arthritis: a pilot study. Arthritis Res. and Ther. 20, 127–15. 10.1186/s13075-018-1624-x 29898765 PMC6001166

[B5] Boyne P. Dunning K. Carl D. Gerson M. Khoury J. Rockwell B. (2016). High-intensity interval training and moderate-intensity continuous training in ambulatory chronic stroke: feasibility study. Phys. Ther. 96 (10), 1533–1544. 10.2522/ptj.20150277 27103222 PMC5046191

[B6] Buchheit M. Laursen P. B. (2013). High-intensity interval training, solutions to the programming puzzle: part I: cardiopulmonary emphasis. Sports Med. 43 (5), 313–338. 10.1007/s40279-013-0029-x 23539308

[B7] Casaña J. Calatayud J. Ezzatvar Y. Vinstrup J. Benítez J. Andersen L. L. (2019). Preoperative high‐intensity strength training improves postural control after TKA: randomized‐controlled trial. Knee Surg. Sports Traumatol. Arthrosc. 27 (4), 1057–1066. 10.1007/s00167-018-5246-2 30361758

[B8] Chen X. Zhang T. Hu X. Wen Z. Lu W. Jiang W. (2024). High-intensity interval training programs *versus* moderate-intensity continuous training for individuals with heart failure: a systematic review and meta-analysis. Archives Phys. Med. rehabilitation 106, 98–112. 10.1016/j.apmr.2024.05.028 38862032

[B9] Costache A. D. Maştaleru A. Leon M. M. Roca M. Gavril R. S. Cosău D. E. (2024). High-intensity interval training vs. medium-intensity continuous training in cardiac rehabilitation programs: a narrative review. Med. Kaunas. Lith. 60 (11), 1875. 10.3390/medicina60111875 PMC11596889 39597060

[B10] Cross M. Smith E. Hoy D. Nolte S. Ackerman I. Fransen M. (2014). The global burden of hip and knee osteoarthritis: estimates from the global burden of disease 2010 study. Ann. Rheumatic Dis. 73 (7), 1323–1330. 10.1136/annrheumdis-2013-204763 24553908

[B11] Cumpston M. Li T. Page M. J. Chandler J. Welch V. A. Higgins J. P. (2019). Updated guidance for trusted systematic reviews: a new edition of the cochrane handbook for systematic reviews of interventions. Cochrane Database Syst. Rev. 10, Ed000142. 10.1002/14651858.ED000142 31643080 PMC10284251

[B12] de Ruiter H. A. van Gorp B. Eijkenboom J. F. A. (2017). Comments on “Effects of high intensity resistance aquatic training on body composition and walking speed in women with mild knee osteoarthritis: a 4-month RCT with 12-month follow-up”. Osteoarthr. Cartil. 25 (11), e17–e18. 10.1016/j.joca.2017.06.011 28803878

[B13] de Zwart A. H. Dekker J. Roorda L. D. van der Esch M. Lips P. van Schoor N. M. (2022). High-intensity *versus* low-intensity resistance training in patients with knee osteoarthritis: a randomized controlled trial. Clin. Rehabil. 36 (7), 952–967. 10.1177/02692155211073039 35331018

[B14] Dunlop D. D. Song J. Hootman J. M. Nevitt M. C. Semanik P. A. Lee J. (2019). One hour a week: moving to prevent disability in adults with lower extremity joint symptoms. Am. J. Prev. Med. 56 (5), 664–672. 10.1016/j.amepre.2018.12.017 30902564 PMC6475497

[B15] Dunlop D. D. Song J. Semanik P. A. Sharma L. Bathon J. M. Eaton C. B. (2014). Relation of physical activity time to incident disability in community dwelling adults with or at risk of knee arthritis: prospective cohort study. Bmj 348, g2472. 10.1136/bmj.g2472 24782514 PMC4004786

[B16] Egger M. Davey Smith G. Schneider M. Minder C. (1997). Bias in meta-analysis detected by a simple, graphical test. BMJ Clin. Res. ed 315 (7109), 629–634. 10.1136/bmj.315.7109.629 PMC2127453 9310563

[B17] Fenton S. A. M. Neogi T. Dunlop D. Nevitt M. Doherty M. Duda J. L. (2018). Does the intensity of daily walking matter for protecting against the development of a slow gait speed in people with or at high risk of knee osteoarthritis? An observational study. Osteoarthr. Cartil. 26 (9), 1181–1189. 10.1016/j.joca.2018.04.015 PMC6098720 29729332

[B18] Foroughi N. Smith R. M. Lange A. K. Baker M. K. Fiatarone Singh M. A. Vanwanseele B. (2011). Lower limb muscle strengthening does not change frontal plane moments in women with knee osteoarthritis: a randomized controlled trial. Clin. Biomech. (Bristol, Avon) 26 (2), 167–174. 10.1016/j.clinbiomech.2010.08.011 20888096

[B19] Gillen J. B. Gibala M. J. (2014). Is high-intensity interval training a time-efficient exercise strategy to improve health and fitness? Appl. Physiology, Nutr. Metabolism 39 (3), 409–412. 10.1139/apnm-2013-0187 24552392

[B20] Higgins J. P. Thompson S. G. (2002). Quantifying heterogeneity in a meta-analysis. Statistics Med. 21 (11), 1539–1558. 10.1002/sim.1186 12111919

[B21] Hua J. Sun L. Teng Y. (2023). Effects of high-intensity strength training in adults with knee osteoarthritis: a systematic review and meta-analysis of randomized controlled trials. Am. J. Phys. Med. and Rehabilitation 102 (4), 292–299. 10.1097/PHM.0000000000002088 36111896

[B22] Husby V. S. Foss O. A. Husby O. S. Winther S. B. (2018). Randomized controlled trial of maximal strength training vs. standard rehabilitation following total knee arthroplasty. Eur. J. Phys. Rehabilitation Med. 54 (3), 371–379. 10.23736/S1973-9087.17.04712-8 28901118

[B23] Hussain S. R. Macaluso A. Pearson S. J. (2016). High-intensity interval training *versus* moderate-intensity continuous training in the prevention/management of cardiovascular disease. Cardiol. Rev. 24 (6), 273–281. 10.1097/CRD.0000000000000124 27548688

[B24] Jones B. Q. Covey C. J. Sineath Jr M. H. (2015). Nonsurgical management of knee pain in adults. Am. Fam. physician 92 (10), 875–883. 26554281

[B25] Keogh J. W. Grigg J. Vertullo C. J. (2018). Is high-intensity interval cycling feasible and more beneficial than continuous cycling for knee osteoarthritic patients? Results of a randomised control feasibility trial. PeerJ 6, e4738. 10.7717/peerj.4738 29761054 PMC5949056

[B26] Keogh J. W. L. Grigg J. Vertullo C. J. (2017). Is home-based, high-intensity interval training cycling feasible and safe for patients with knee osteoarthritis? Study protocol for a randomized pilot study. Orthop. J. Sports Med. 5 (3), 2325967117694334. 10.1177/2325967117694334 28451599 PMC5400173

[B27] Lane N. E. Hochberg M. C. Nevitt M. C. Simon L. S. Nelson A. E. Doherty M. (2015). OARSI clinical trials recommendations: design and conduct of clinical trials for hip osteoarthritis. Osteoarthr. Cartil. 23 (5), 761–771. 10.1016/j.joca.2015.03.006 25952347

[B28] Liu C.-J. Latham N. (2010). Adverse events reported in progressive resistance strength training trials in older adults: 2 sides of a coin. Archives Phys. Med. Rehabilitation 91 (9), 1471–1473. 10.1016/j.apmr.2010.06.001 20801270

[B29] Lo G. H. Schneider E. Driban J. B. Price L. L. Hunter D. J. Eaton C. B. et al. (2018). Periarticular bone predicts knee osteoarthritis progression: data from the osteoarthritis Initiative 2018 (Elsevier). 10.1016/j.semarthrit.2018.01.008 PMC6853601 29449014

[B30] Messier S. P. Mihalko S. L. Beavers D. P. Nicklas B. J. DeVita P. Carr J. J. (2021). Effect of high-intensity strength training on knee pain and knee joint compressive forces among adults with knee osteoarthritis: the START randomized clinical trial. Jama 325 (7), 646–657. 10.1001/jama.2021.0411 33591346 PMC7887656

[B31] Oliveira A. Fidalgo A. Farinatti P. Monteiro W. (2024). Effects of high-intensity interval and continuous moderate aerobic training on fitness and health markers of older adults: a systematic review and meta-analysis. Archives Gerontology Geriatrics 124, 105451. 10.1016/j.archger.2024.105451 38718488

[B32] O’Sullivan O. (2024) “Osteoarthritis: pathophysiology and classification of a common disabling condition,” in The palgrave encyclopedia of disability. Springer, 1–11.

[B33] Page M. J. McKenzie J. E. Bossuyt P. M. Boutron I. Hoffmann T. C. Mulrow C. D. (2021). The PRISMA 2020 statement: an updated guideline for reporting systematic reviews. BMJ Clin. Res. ed 372, n71. 10.1136/bmj.n71 PMC8005924 33782057

[B34] Regnaux J. P. Lefevre‐Colau M. M. Trinquart L. Nguyen C. Boutron I. Brosseau L. (2015). High‐intensity *versus* low‐intensity physical activity or exercise in people with hip or knee osteoarthritis. Cochrane Database Syst. Rev. 2015 (10), CD010203. 10.1002/14651858.CD010203.pub2 26513223 PMC9270723

[B35] Ross L. M. Porter R. R. Durstine J. L. (2016). High-intensity interval training (HIIT) for patients with chronic diseases. J. Sport health Sci. 5 (2), 139–144. 10.1016/j.jshs.2016.04.005 30356536 PMC6188712

[B36] Roxburgh B. H. Campbell H. A. Cotter J. D. Reymann U. Williams M. J. A. Gwynne-Jones D. (2024). Upper-limb high-intensity interval training or passive heat therapy to optimize cardiorespiratory fitness prior to total hip or knee arthroplasty: a randomized controlled trial. Arthritis Care and Res. 76 (3), 393–402. 10.1002/acr.25238 37728076

[B37] Sandstad J. Stensvold D. Hoff M. Nes B. M. Arbo I. Bye A. (2015). The effects of high intensity interval training in women with rheumatic disease: a pilot study. Eur. J. Appl. Physiology 115, 2081–2089. 10.1007/s00421-015-3186-9 26013051

[B38] Sayer A. A. Robinson S. M. Patel H. P. Shavlakadze T. Cooper C. Grounds M. D. (2013). New Horizons in the pathogenesis, diagnosis and management of sarcopenia. Age Ageing 42 (2), 145–150. 10.1093/ageing/afs191 23315797 PMC3575121

[B39] Su C. Huang L. Tu S. Lu S. (2024). Different intensities of aerobic training for patients with type 2 diabetes mellitus and knee osteoarthritis: a randomized controlled trial. Front. Endocrinol. 15, 1463587. 10.3389/fendo.2024.1463587 PMC11402742 39286270

[B40] Tanaka R. Ozawa J. Kito N. Moriyama H. (2015). Does exercise therapy improve the health-related quality of life of people with knee osteoarthritis? A systematic review and meta-analysis of randomized controlled trials. J. Phys. Ther. Sci. 27 (10), 3309–3314. 10.1589/jpts.27.3309 26644699 PMC4668190

[B41] Thomsen R. S. Nilsen T. I. L. Haugeberg G. Bye A. Kavanaugh A. Hoff M. (2018). Effect of high-intensity interval training on cardiovascular disease risk factors and body composition in psoriatic arthritis: a randomised controlled trial. RMD open 4 (2), e000729. 10.1136/rmdopen-2018-000729 30402265 PMC6203095

[B42] Thudium C. S. Engstrøm A. Bay-Jensen A. C. Frederiksen P. Jansen N. De Zwart A. (2023). Cartilage tissue turnover increases with high-compared to low-intensity resistance training in patients with knee OA. Arthritis Res. and Ther. 25 (1), 22. 10.1186/s13075-023-03000-2 36765372 PMC9912672

[B43] Torstensen T. A. Østerås H. LoMartire R. Rugelbak G. M. Grooten W. J. A. Äng B. O. (2023). High- *versus* low-dose exercise therapy for knee osteoarthritis: a randomized controlled multicenter trial. Ann. Intern. Med. 176 (2), 154–165. 10.7326/M22-2348 36689746

[B44] Uthman O. A. van der Windt D. A. Jordan J. L. Dziedzic K. S. Healey E. L. Peat G. M. (2014). Exercise for lower limb osteoarthritis: systematic review incorporating trial sequential analysis and network meta-analysis. Br. J. sports Med. 48 (21), 1579. 10.1136/bjsports-2014-5555rep 25313133

[B45] Waller B. Munukka M. Rantalainen T. Lammentausta E. Nieminen M. T. Kiviranta I. (2017). Effects of high intensity resistance aquatic training on body composition and walking speed in women with mild knee osteoarthritis: a 4-month RCT with 12-month follow-up. Osteoarthr. Cartil. 25 (8), 1238–1246. 10.1016/j.joca.2017.02.800 28263901

[B46] Wang Z. Wang J. (2024). The effects of high-intensity interval training *versus* moderate-intensity continuous training on athletes' aerobic endurance performance parameters. Eur. J. Appl. Physiology 124 (8), 2235–2249. 10.1007/s00421-024-05532-0 38904772

[B47] Yoshimura N. Muraki S. Oka H. Tanaka S. Kawaguchi H. Nakamura K. (2012). Accumulation of metabolic risk factors such as overweight, hypertension, dyslipidaemia, and impaired glucose tolerance raises the risk of occurrence and progression of knee osteoarthritis: a 3-year follow-up of the ROAD study. Osteoarthr. Cartil. 20 (11), 1217–1226. 10.1016/j.joca.2012.06.006 22796312

