# Supplementary material for: Effect of high-intensity training on improving knee flexion strength and quality of life in patients with knee osteoarthritis: a systematic review and meta-analysis of randomized controlled trials
Source: Front Physiol. 2025 Jun 27;16:1561697. doi: 10.3389/fphys.2025.1561697 (PMC12245925; doi:10.3389/fphys.2025.1561697)
Supplement: Supplementary file 1 [file DataSheet1.pdf]

Supplementary materials

Table S1 Literature Search Strategy

Pubmed-85
(((("Exercise"[Mesh]) OR ((((((((((((Exercises) OR (Physical Exercise)) OR (Physical Exercises)) OR (Physical Activity)) OR (Physical Activities)) OR (Aerobic Exercise)) OR (Aerobic Exercises)) OR (Isometric Exercises)) OR (Isometric Exercise)) OR (Acute Exercise)) OR (Acute Exercises)) OR (Exercise Training)) OR (Exercise Trainings))) AND ((high intensity) OR (high-intensity))) AND (("Osteoarthritis, Knee"[Mesh]) OR ((((Knee Osteoarthritides) OR (Knee Osteoarthritis)) OR (Osteoarthritis of the Knee)) OR (Osteoarthritis of Knee)))) AND (Random*)
Embase-69
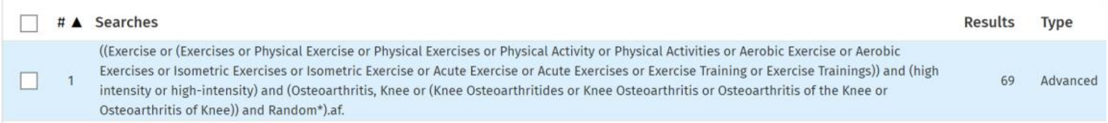 # ▲ Searches — Results — Type<br>1 — ((Exercise or (Exercises or Physical Exercise or Physical Exercises or Physical Activity or Physical Activities or Aerobic Exercise or Aerobic Exercises or Isometric Exercises or Isometric Exercise or Acute Exercise or Acute Exercises or Exercise Training or Exercise Trainings)) and (high intensity or high-intensity) and (Osteoarthritis, Knee or (Knee Osteoarthritides or Knee Osteoarthritis or Osteoarthritis of the Knee or Osteoarthritis of Knee)) and Random*).af. — 69 — Advanced
Cochrane-81
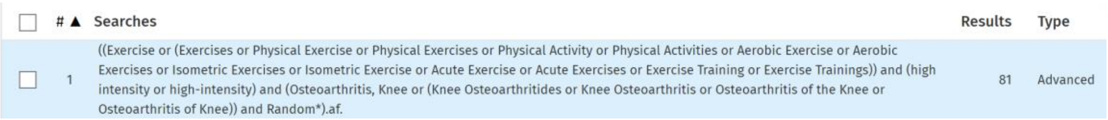 # ▲ Searches — Results — Type<br>1 — ((Exercise or (Exercises or Physical Exercise or Physical Exercises or Physical Activity or Physical Activities or Aerobic Exercise or Aerobic Exercises or Isometric Exercises or Isometric Exercise or Acute Exercise or Acute Exercises or Exercise Training or Exercise Trainings)) and (high intensity or high-intensity) and (Osteoarthritis, Knee or (Knee Osteoarthritides or Knee Osteoarthritis or Osteoarthritis of the Knee or Osteoarthritis of Knee)) and Random*).af. — 81 — Advanced
WOS-214
((((Exercise) OR ((((((((((((Exercises) OR (Physical Exercise)) OR (Physical Exercises)) OR (Physical Activity)) OR (Physical Activities)) OR (Aerobic Exercise)) OR (Aerobic Exercises)) OR (Isometric Exercises)) OR (Isometric Exercise)) OR (Acute Exercise)) OR (Acute Exercises)) OR (Exercise Training)) OR (Exercise Trainings))) AND ((high intensity) OR (high-intensity))) AND ((Osteoarthritis, Knee) OR ((((Knee Osteoarthritides) OR (Knee Osteoarthritis)) OR (Osteoarthritis of the Knee)) OR (Osteoarthritis of Knee)))) AND (Random*) (Topic)

Figure S1: Sensitivity analysis of (A) 6-MWT, (B) KOOS pain, (C) knee flexion strength, (D) knee extension strength.

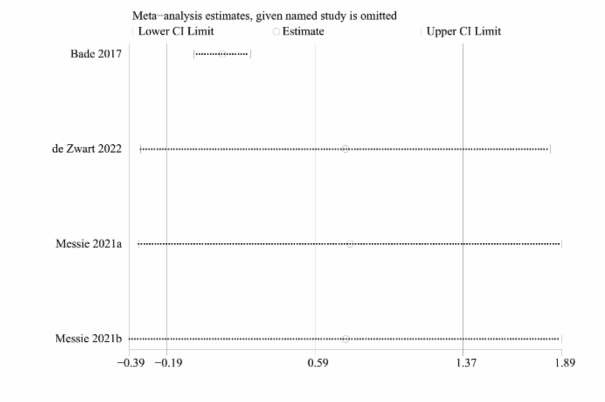

Study omitted	Estimate	[95% Conf.	Interval]
Bade 2017	.09850792	-.05181329	.24882913
de Zwart 2022	.74860275	-.3328594	1.8300648
Messie 2021a	.77365905	-.34313905	1.8904572
Messie 2021b	.74905616	-.39205152	1.8901638
Combined	.58815847	-.19288492	1.3692019

A

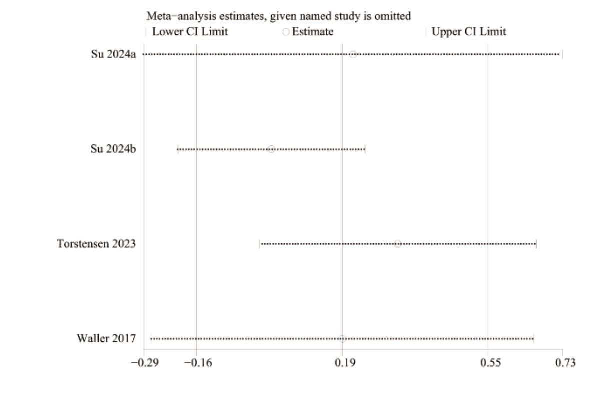

Study omitted	Estimate	[95% Conf.	Interval]
Su 2024a	.21907525	-.28900346	.72715396
Su 2024b	.02094398	-.20622461	.24811257
Torstensen 2023	.32725981	-.00828315	.66280276
Waller 2017	.19318928	-.27005401	.65643257
Combined	.19294433	-.15970783	.54559649

B

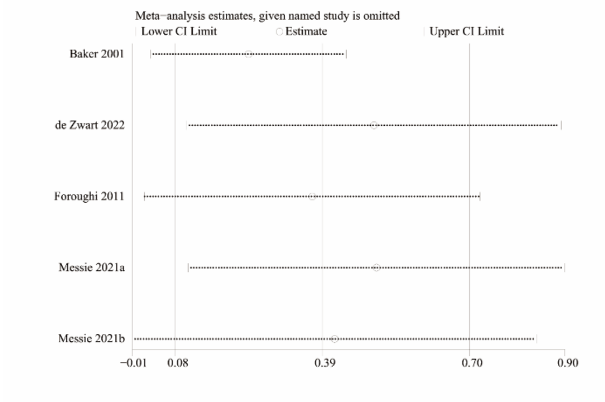

Study omitted	Estimate	[95% Conf.	Interval]
Baker 2001	.23221289	.02642905	.43799672
de Zwart 2022	.49595597	.10230765	.88960427
Foroughi 2011	.36639482	.01380847	.71898115
Messie 2021a	.50142562	.10552029	.89733094
Messie 2021b	.41318777	-.01199067	.83836621
Combined	.38687685	.07764001	.69611368

C

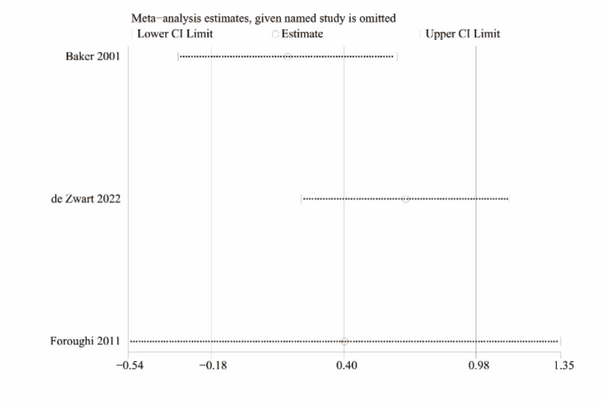

Study omitted	Estimate	[95% Conf.	Interval]
Baker 2001	.15516084	-.32600862	.63633031
de Zwart 2022	.6714474	.21498874	1.1279061
Foroughi 2011	.40410876	-.54388446	1.3521019
Combined	.40043352	-.17876073	.97962777

D
